# SELF-PERCEPTIONS OF AGING AND DAILY SOCIAL INTERACTIONS

**DOI:** 10.1093/geroni/igae098.2102

**Published:** 2024-12-31

**Authors:** Lydia Ong, Patrick Klaiber, Nicole Stuart, Anita DeLongis, Nancy Sin

**Affiliations:** University of British Columbia, Vancouver, British Columbia, Canada; Tilburg University, Tilburg, Noord-Brabant, Netherlands; University of British Columbia, Vancouver, British Columbia, Canada; University of British Columbia, Vancouver, British Columbia, Canada; University of British Columbia, Vancouver, British Columbia, Canada

## Abstract

Age stereotypes can become internalized across the lifespan to influence one’s self-perceptions of aging (SPA). Stereotype embodiment theory suggests behavioral and stress response pathways through which SPA influence downstream health outcomes. Previous research has shown negative SPA predict lower social engagement, but little is known about whether this mechanism is evident at the daily level. Thus, the current study uses ecological momentary assessment over 14 days to investigate whether more negative SPA are associated with fewer interpersonal stressors and positive social interactions, and whether SPA attenuate differences in positive and negative affect on moments with versus without these events. Participants were 224 adults from British Columbia, Canada (ages 25-89, M = 46, 71% women). Multiple regression and three-level multilevel models controlled for demographic factors, depressive symptoms, and health conditions. Results showed that SPA did not predict exposure to interpersonal stressors and positive social interactions. People with more negative SPA had larger differences in negative affect on moments with versus without interpersonal stressors (simple slope for -1 SD SPA: b =.99, SE =.13, p <.001), compared to those with less negative SPA (simple slope for +1 SD SPA: b =.60, SE =.14, p <.001). SPA did not moderate associations between interpersonal stressors and positive affect, or between positive social interactions and positive and negative affect. Findings indicate that people with more negative SPA do not show a lack of daily social engagement but display greater negative affective stress responses, which have implications for downstream health.

